# Application of novel analytical ultracentrifuge analysis to solutions of fungal mannans

**DOI:** 10.1007/s00249-016-1159-5

**Published:** 2016-07-21

**Authors:** Richard B. Gillis, Gary G. Adams, David T. M. Besong, Eva Machová, Anna Ebringerová, Arthur J. Rowe, Stephen E. Harding, Trushar R. Patel

**Affiliations:** 1 Faculty of Medicine and Health Sciences, Queens Medical Centre, University of Nottingham, Nottingham, NG7 2UH UK; 2 National Centre for Macromolecular Hydrodynamics, School of Biosciences, University of Nottingham, Sutton Bonington, LE12 5RD UK; 3 Functional Nanomaterials Lab, King Abdullah University of Science and Technology (KAUST), Thuwal, 23955-6900 Kingdom of Saudi Arabia; 4 Centre of Glycomics, Institute of Chemistry, Slovak Academy of Sciences, Bratislava, 84548 Slovakia; 5 Department of Chemistry and Biochemistry, Alberta RNA Research and Training Institute, University of Lethbridge, 4401 University Drive, Lethbridge, AB T1K 3M4 Canada

**Keywords:** AUC, Extended Fujita approach, MULTISIG, SEDFIT-MSTAR, Sedimentation

## Abstract

Polysaccharides, the most abundant biopolymers, are required for a host of activities in lower organisms, animals, and plants. Their solution characterization is challenging due to their complex shape, heterogeneity, and size. Here, recently developed data analysis approaches were applied for traditional sedimentation equilibrium and velocity methods in order to investigate the molar mass distribution(s) of a subtype of polysaccharide, namely, mannans from four *Candida* spp. The molecular weight distributions of these mannans were studied using two recently developed equilibrium approaches: SEDFIT-MSTAR and MULTISIG, resulting in corroboratory distribution profiles. Additionally, sedimentation velocity data for all four mannans, analyzed using ls-g*(*s*) and Extended Fujita approaches, suggest that two of the fungal mannans (FM-1 and FM-3) have a unimodal distribution of molecular species whereas two others (FM-2 and FM-4) displayed bi-modal and broad distributions, respectively: this demonstrates considerable molecular heterogeneity in these polysaccharides, consistent with previous observations of mannans and polysaccharides in general. These methods not only have applications for the characterization of mannans but for other biopolymers such as polysaccharides, DNA, and proteins (including intrinsically disordered proteins).

## Introduction

**Fig. 1:**
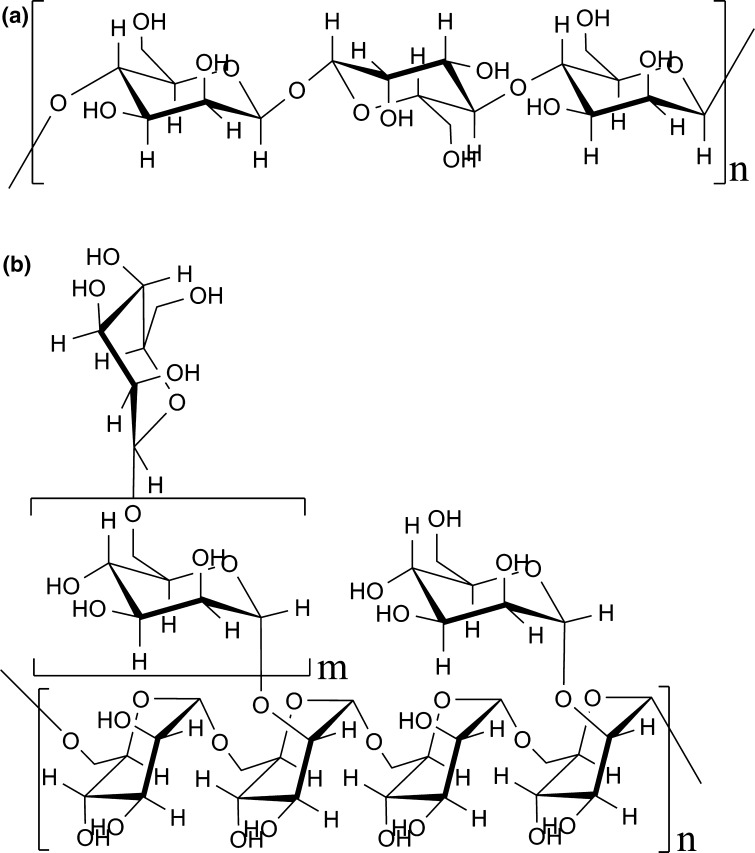
Chemical structure of mannan from **a** plant sources and **b** fungal sources

Mannans are polysaccharides containing d-mannose, and are found as cell wall components in bacteria, fungi, (moulds and yeast) and plants. Pure mannan is uncommon in plants but it is one of the major components of the yeast cell wall together with glucan, chitin, and protein such as mannoprotein. Mannans have different kinds of structures in various organisms. Figure 1a describes the structure of plant mannan, which has a backbone of linear chains made up of β(1 → 4)-linked Mannopyrosyl (Man*p*) residues (Tombs and Harding 1998). Plant mannans occur in the cell walls as heteropolysaccharides, i.e., glucomannans, galactoglucomannans, and galactomannans (Ebringerova et al. 2005). Generally, *Candida* spp. mannans have an α(1 → 6) linked backbone (Fig. 1b) substituted mostly at *O*-2 by different number of linear or branched side oligomannosyl chains composed of α(1 → 2), α(1 → 3) and α(1 → 6) links with or without terminal β(1 → 2) linkages (Shibata et al. 1992, 1996, 2003, 2007). It was also reported that there are subtle variations in the linkage or number of mannose residues in side chains of *Candida* spp. mannan molecules (Nelson et al. 1991). *C. tropicalis* mannan is composed of an α(1 → 6)-linked backbone substituted only with β(1 → 2) linked and α(1 → 2) linked Man*p* units without α(1 → 3) linked ones observed in *C. albicans* mannan (Kobayashi et al. 1994; Suzuki et al. 1997). *C. dubliniensis* has been isolated from the HIV-positive individuals in the beginning of 1990 [(Pujol et al. 2004) and references cited therein] and later grouped separately from *C. albicans* by Sullivan et al. (1995) Mannan from *C. dubliniensis* (Ližičárová et al. 2005), as well as mannan from *C. parapsilosis* (Shibata et al. 1995), have very similar structures to that of *C. albicans*.

The anti-tumor activity of polysaccharides was reported by Diller (1947), followed by several other authors who suggested that mannans are potent anticancer agents (Mankowski et al. 1957; Bradner and Clarke 1959; Kamasuka et al. 1968; Oka et al. 1969; Suzuki et al. 1969; Ukai et al. 1983; Peng et al. 2003). It is also critical to characterize these polysaccharides because certain factors, such as polymer length (proportionally linked to molecular weight), can impact their potency, for example their antigenicity (Kabat and Bezer 1958).

This article is focused on the characterization of fungal mannans using analytical ultracentrifugation (AUC)—a well-established, matrix-free method for the determination of molar mass and sedimentation coefficients. In addition, recently presented analysis techniques have been utilized to determine the molar mass distribution of fungal mannans, along with providing distributions of sedimentation coefficients.

## Methods

Four mannan samples, FM-1 (*Candida tropicalis* CCY 29-7-6), FM-2 (*C. dubliniensis* CCY 29-177-1), FM-3 (*C. albicans* CCY 29-3-32), and FM-4 (*C. parapsilosis* CCY 29-20-1) were prepared from the above-mentioned yeast strains obtained from Culture Collection of Yeast (Institute of Chemistry, Bratislava, Slovakia). The mannans were prepared from fresh yeast biomass as described by Bystrický et al. (2003). Under the used strong alkaline conditions, nearly all covalently linked protein was split from the mannoproteins. FM-3 contained 0.4 % nitrogen whereas FM-1, FM-2, and FM-4 only traces.

### Sample preparation

Samples were dissolved in phosphate buffered saline (PBS, pH 7.0, *I* = 0.1 M), made with 0.05 M sodium chloride, and 0.05 M phosphate salts (dibasic sodium and potassium dihydrogen) in screw-capped tubes with constant stirring at low speed. During this period, the temperature was raised to 80.0 °C for 10 min to obtain maximum solubility. Stirring continued overnight at room temperature at low speed. Samples were subsequently centrifuged at 10,000 rpm (11,600×*g*) for 15 min (Beckman L8-55 M Ultracentrifuge). Concentrations of stock solutions were measured using a differential refractometer (Atago DD-5, Jencons Scientific) and a refractive index increment of 0.171 ml g^−1^ (Mueller et al. 2000).

### Molar mass determination using sedimentation equilibrium

All mannan samples were subjected to sedimentation equilibrium analytical ultracentrifugation (AUC-SE) experiments using the Optima XL-I Analytical Ultracentrifuge (Beckman Instruments, Palo Alto, CA). Double-sector carbon-filled epoxy 12-mm path-length centerpieces, loaded into aluminium housings and sealed with sapphire windows, were used to load solvent (90 μl), and sample (80 μl) at 1.0 mg ml^−1^. Cells were loaded into eight-hole titanium rotor (An50Ti) and placed in the centrifuge. Samples were centrifuged at 16,000 rpm (~20,600×*g*) at (20.0 ± 0.1) °C. Scans were taken using Rayleigh interference optics once every hour until equilibrium was achieved.

Data analysis was performed using two independent algorithms. SEDFIT-MSTAR (Schuck et al. 2014), which utilizes a smart-smooth method to fit the raw data curve, and M* function (Harding et al. 1992), to provide the weight average of the entire distribution of molar masses:

1 $$M^{*} \left( r \right) = \frac{J\left( r \right) - J}{{kJ_{\text{m}} \left( {r^{2} - r_{\text{m}}^{2} } \right) + 2k\int\limits_{{r_{\text{m}} }}^{r} {\left( {J\left( r \right) - J} \right)r} \cdot {\text{d}}r}}$$

2 $$k = \frac{{\left( {1 - \overline{v} \rho } \right)\omega^{2} }}{2RT}$$

In Eqs. (1) and (2), *r* is the radial distance from the center of rotation, *r*_m_ the corresponding value at the meniscus, *J* is the concentration in fringe displacement units, and *J*_m_ the corresponding value at the meniscus. *ῡ* is the partial specific volume, *ρ* is the solvent density, *ω* is the angular velocity of the rotor, *R* is the gas constant and *T* is the absolute temperature.

MULTISIG was then used to fit the relative concentration proportions of 17 molar masses, logarithmically spaced to achieve a tenfold range, to yield a molar mass distribution (Gillis et al. 2013). The total (fringe) concentration at a set radial position is given by:

3 $$J\left( r \right) = \sum\limits_{i = 1}^{i = 17} {J_{\text{ref}} \exp } \left\{ {0.5\left( {0.5kM_{i} 1.15^{{\left( {i - 1} \right)}} \left( {r^{2} - r_{\text{m}}^{2} } \right)} \right)} \right\} + E$$

where *M*_*i*_ is the species molar mass, *J*_ref_ is the reference concentration (typically the concentration at the consensus hinge point), and *E* is the baseline. MULTISIG/RADIUS was used to apply this procedure to radial positions along the column length of solution. Both methods were performed in pro Fit™ (QuantumSoft, Switzerland).

### Sedimentation velocity analysis

Sedimentation coefficient distributions of mannans were measured using sedimentation velocity (AUC-SV) in the analytical ultracentrifuge. Solvent (400 μl) and sample (390 μl, 1.0 mg ml^−1^) were loaded into similarly constructed cells as for AUC-SE. Cells were centrifuged in the Beckman Optima XL-I analytical ultracentrifuge at 40,000 rpm (~130,000×*g*) at (20.0 ± 0.1) °C. Data were analyzed using least squares apparent distributions of sedimentation coefficients (ls-g*(*s*) vs. *s*) from SEDFIT (Schuck and Rossmanith 2000), and curve fitting module MULTIG in pro Fit™ (QuantumSoft, Switzerland). Weight-average sedimentation coefficients (*s*) for particular components were corrected to standard solvent conditions (density and viscosity of water at 20.0 °C) to yield *s*_20,*w*_ (S), using SEDNTERP (Laue et al. 1992), and a *ῡ* of 0.625 ml g^−1^ (Gray and Ballou 1971). The *s*_20,*w*_ was measured at a range of concentrations (0.2–2 mg ml^−1^) for all samples and extrapolation performed for each to zero concentration to obtain $s_{20,w}^{0}$ to eliminate the effects of non-ideality.

## Results and discussion

### Molar mass distribution

Mannan samples, both dialyzed and undialyzed, were probed for their molar masses using sedimentation equilibrium. There was little observable difference in terms of their molar masses between dialyzed and undialyzed samples (data not shown), suggesting the sample was of high purity. Results presented below are from dialyzed samples.

### SEDFIT-MSTAR

**Fig. 2:**
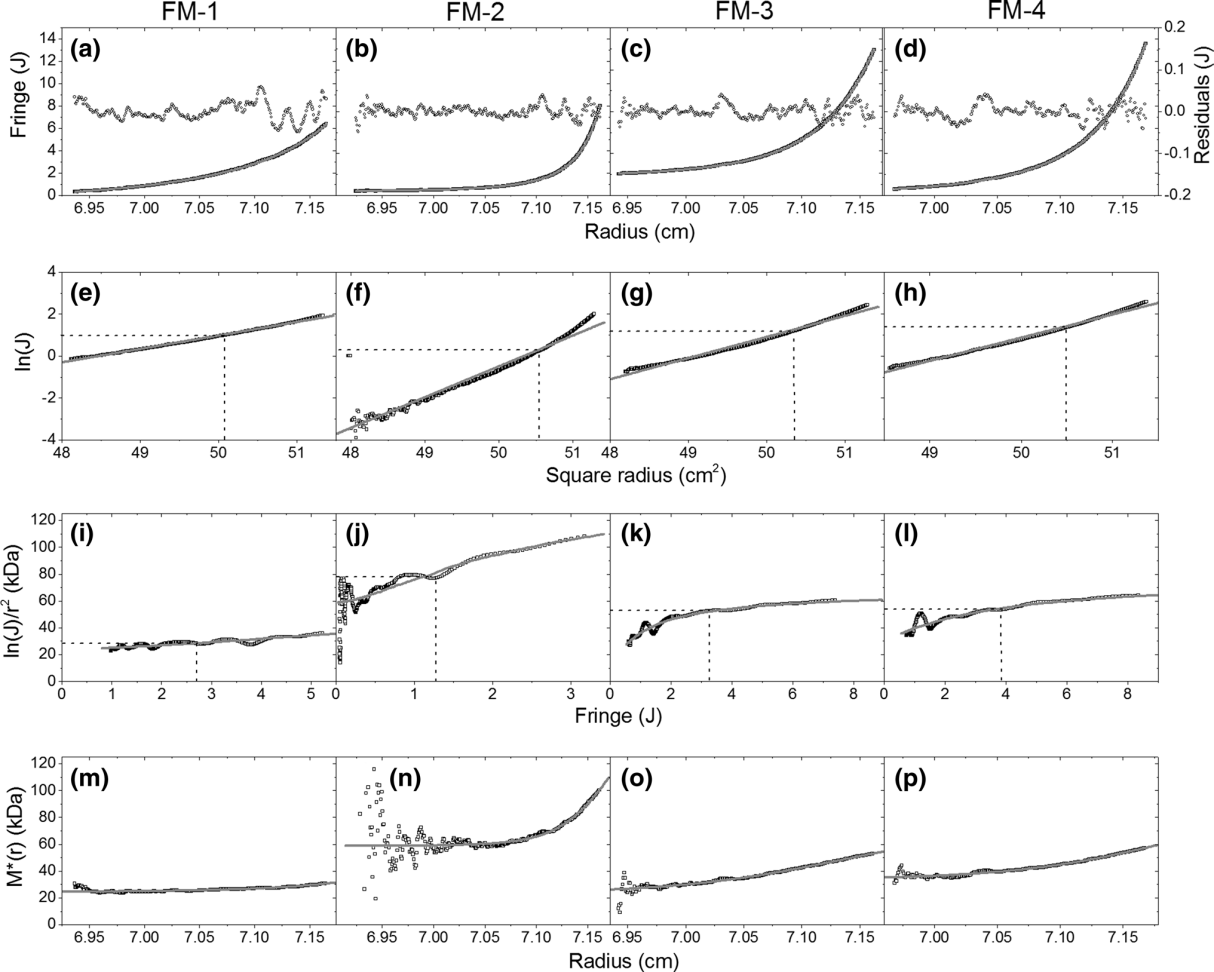
Output from SEDFIT-MSTAR of FM1-4 (*left* to *right*). **a**–**d** Fringe displacement (**j**) vs. radius with residual between raw data (*hollow square*) and fit (*grey line*); **e**–**h** natural logarithm of baseline-corrected fringe displacement *J* vs. square radius; **i**–**l** differential of **e**–**h** yielding apparent molar mass vs. concentration; **m**–**p** M*(*r*) algorithm extrapolating to cell base

SEDFIT-MSTAR fitted the raw fringe displacement data using the smart-smooth analysis (Fig. 2a–d) with results shown in Table 1. The grey lines represent the result of the fitted parameters. Residuals show no overall trend and do not deviate beyond 0.1 fringes in all samples. The natural logarithm of (baseline-corrected) fringe displacement (*J*) versus the square of the radius (Fig. 2e–h) shows a near-straight line but with small positive curvature, particularly in FM-2. This indicates polydispersity. The differential of Fig. 2e–h yields Fig. 2i–l representing point-average, apparent *M*_*w*_(*r*) as a function of the concentration *c*(*r*) across the cell. Positive slopes suggest polydispersity, especially in FM-2 (Fig. 2j) where values for the *M*_*w*_(*r*) appear to fall significantly as zero concentration is approached. This result is consistent with results from ls-g*(*s*) analysis suggesting a bimodal distribution; thus the lower radial positions in the cell would contain a high proportion of the low-molar-mass species.

**Table 1:** Weight average and *z*-average molar mass estimates from AUC-SE of fungal mannans using SEDFIT-MSTAR

Sample	×10^−3^ *w*-average molar mass^a^ (g mol^−1^)	×10^−3^ *w*-average molar mass^b^ (g mol^−1^)	×10^−3^ *z*-average molar mass^b^ (g mol^−1^)	PDI^b^ (z/w)
FM-1	30.2	31.5	41.6	1.32
FM-2	83.3	112	140	1.25
FM-3	56.1	54.8	64.7	1.18
FM-4	58.5	59.6	71.6	1.20

Loading concentrations were approximately 1 mg ml^−1^. Polydispersity index (PDI) measured as a ratio of *z*-average and *w*-average from SEDFIT-MSTAR *c*(*M*) fit

^a^From the Consensus Hinge Point (CHP) method

^b^From extrapolation of M*(*r*) to the cell base (Eq. 1, Fig. 2) and SEDFIT-MSTAR fit

The MSTAR algorithm yielded weight-average molar masses (*M*_*w*_) ranging from 3.1 to 11.2 × 10^4^ g mol^−1^ through the extrapolation of the M*(*r*) to the base of the cell (Fig. 2m–p) and *z*-average molar masses (*M*_*z*_) ranging from 4.1 to 14 × 10^4^ g mol^−1^. The ratio of *M*_*z*_/*M*_*w*_ provides the polydispersity index (PDI) ranging from 1.2 to 1.3, typical for a polydisperse system. Consensus hinge points (CHP—the point at which, during the approach to equilibrium, the concentration does not change significantly over time; as well as an indication of the loading concentration of the sample) were measured as an internal check of the rigor of the MSTAR analysis. Hinge points are indicated in Fig. 2 by dashed lines and results are presented, with the other results from MSTAR and *c*(*M*) analysis, in Table 1. CHP results do not deviate greatly from the MSTAR and *c*(*M*) results, suggesting that non-ideality had no significant impact on the overall analysis (Schuck et al. 2014).

### MULTISIG

**Fig. 3:**
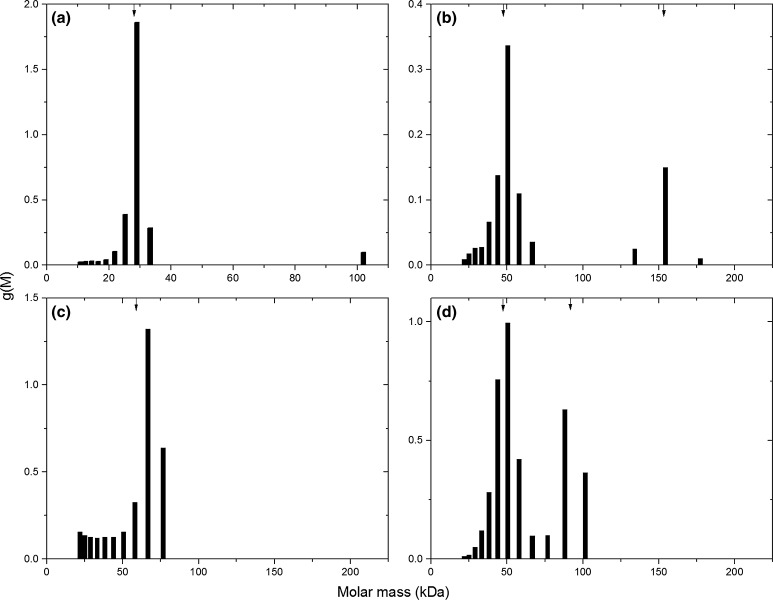
MULTISIG output of **a** FM-1; **b** FM-2; **c** FM-3; and **d** FM-4, including 17 discrete molar mass values. *Arrows* represent weight-average of peak(s)

MULTISIG approximates the real solute distribution present by a series of ‘concentration’ coefficients attached to terms in reduced molar mass value, which are logarithmically spaced, thus yielding a distribution (*g*(*M*) vs. *M*) (Gillis et al. 2013). These are presented in Fig. 3. FM-2 and FM-4 both show two peaks, which is consistent with ls-g*(*s*) distributions. Number, weight, and *z*-average molar masses, along with polydispersity indices, are shown in Table 2. Estimates are also made in Table 2 for peak molar masses and relative concentrations.

**Table 2:** Output from MULTISIG of fungal mannans measured at a loading concentration of 1 mg ml^−1^

Sample	Peak	Fraction (%)	×10^−3^ *n*-average molar mass (g mol^−1^)	×10^−3^ *w*-average molar mass (g mol^−1^)	×10^−3^ *z*-average molar mass (g mol^−1^)	PDI (*z*/*w*)
FM-1	1	97	28.8	29.7	30.3	1.02
	Total	100	29.5 (±0.2)	32.3 (±0.1)	39.2 (±0.0)	1.22
FM-2	1	81	48.5	50.8	52.8	1.04
	2	19	162	163	163	1.00
	Total	100	56.3 (±1.0)	67.2 (±0.2)	101 (±0.0)	1.39
FM-3	1	80	69.0	70.4	71.5	1.02
	Total	100	54.2 (±0.6)	62.3 (±0.1)	67.6 (±0.0)	1.09
FM-4	1	82	49.1	50.8	52.3	1.03
	2	28	96.9	97.6	98.3	1.01
	Total	100	57.1 (±0.2)	64.2 (±0.0)	72.3 (±0.0)	1.13

Averages (*n*, *w*, *z*) and polydispersity index (PDI, *z*/*w*) produced from both the overall distribution (‘Total’) and from individual peaks. Standard error of the mean represented by* parentheses*

### MULTISIG/RADIUS

**Fig. 4:**
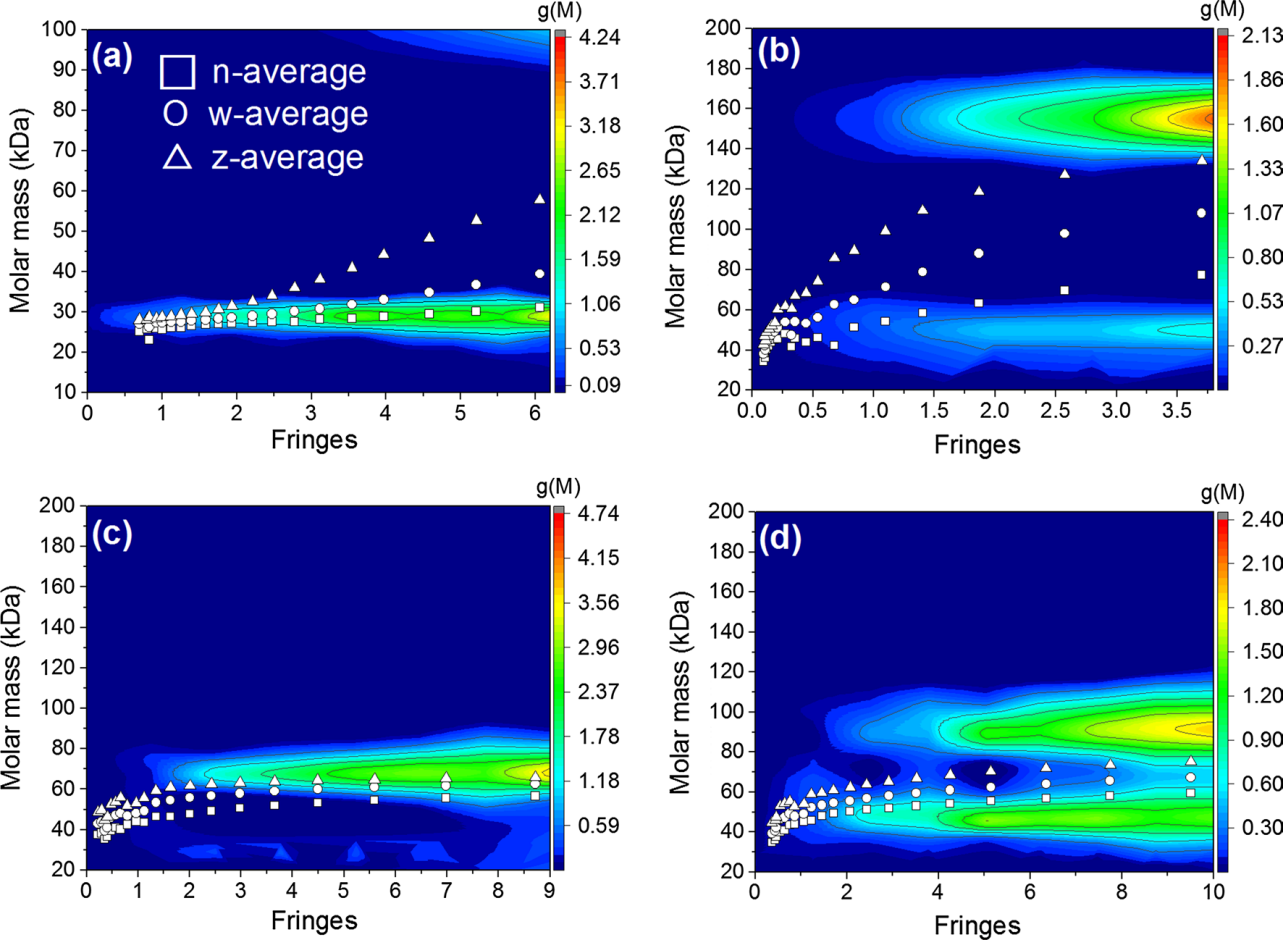
MULTISIG/RADIUS output of **a** FM-1; **b** FM-2; **c** FM-3; and **d** FM-4. 17 discrete molar masses along 20 points of the cell. Number (*square*), weight (*circle*), and z-average (*triangle*) molar masses overlaid

Whilst MULTISIG fits 17 discrete species at a selected radial position (in this investigation, the CHP, i.e., the point in the curve where the concentration does not change over the approach to equilibrium), MULTISIG/RADIUS provides this estimate at 20 points along the range of the cell. The baseline was fixed based on ten iterations from the previous MULTISIG analysis. Results yielded number, weight, and z-average reduced molar masses plotted against the concentration range of the cell and a 3D contour plot of *g*(*M*) vs. *M* vs. concentration range (Fig. 4). Number, weight, and *z*-average molar masses produced similar trends to those found from SEDFIT-MSTAR (Fig. 2i–l), which is unsurprising since these plots should provide equivalent information, despite being independently calculated. The contour plot indicates that the twin-peak distributions found in FM-2 and FM-4 are present throughout the cell context with little change in relative strength—with the exception of FM-2, which has a low proportion of the high-*M*_*w*_ species at the top of the cell. This can be explained by the high* g* force depleting the high-*M*_*w*_ component at the meniscus. PDI (*z*/*w*) is also consistent to within a reasonable margin of error compared to SEDFIT-MSTAR values, although a direct comparison is difficult due to the peak-identifying nature of MULTISIG and the whole-solution evaluation from SEDFIT-MSTAR.

Weight averages present themselves directly in-between the two peaks of FM-2 and FM-4, whereas they lie closer to the main peaks present in FM-1 and FM-3; however, they are slightly skewed towards small amounts of smaller (FM-3) or larger (FM-1) material. It is unclear whether these smaller or larger species are indicative of impurities or algorithmic anomalies, however the averages are similar to those found from the independent SEDFIT-MSTAR analysis approach.

### Sedimentation coefficient distribution

**Fig. 5:**
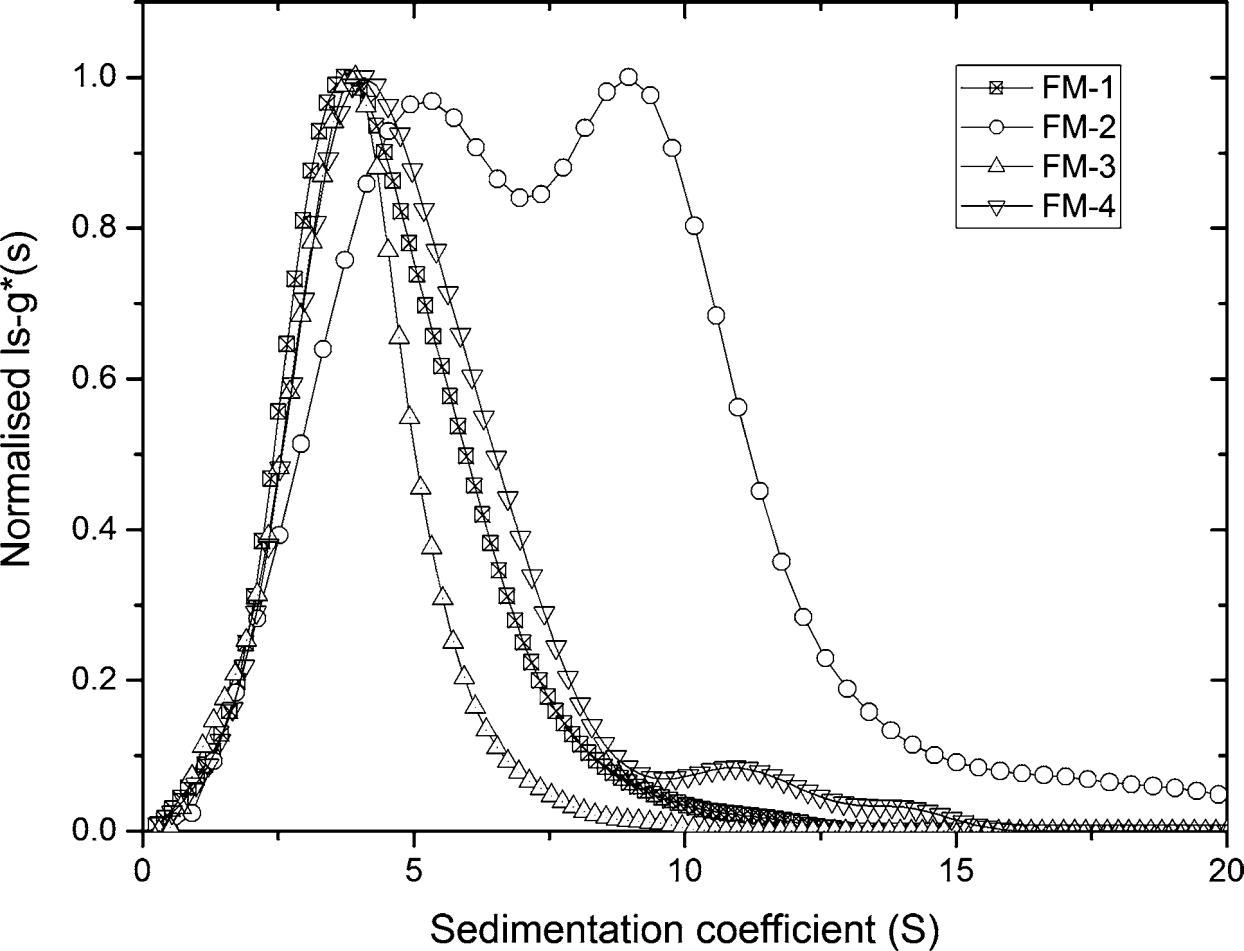
Normalized ls-g*(*s*) vs. sedimentation coefficient of fungal mannans at highest concentration (~2 mg ml^−1^)

The ls-g*(*s*) profiles for FM-1, FM-2, FM-3 and FM-4 are shown in Fig. 5, at approximately 2 mg ml^−1^, as normalized distributions. The profiles indicate that FM-1 and FM-3 yielded a single distribution at ~4 S with a small degree of larger material in the high-sedimentation range (up to 20 S). FM-4 peaks at ~4 S and has a larger proportion of faster-sedimenting material, particularly at 11 and 14 S. FM-2 yielded a two-peak distribution at ~4 and ~9 S.

The concentration for each component was re-calculated based on the initial loading concentration, and amount of mannan fraction present, and used to calculate the $s_{20,w}^{0}$ (S) from *s*_20,*w*_. Extrapolations were performed using the reciprocal sedimentation coefficients as shown in Fig. 5 as before (Patel et al. 2006, 2008). The Gralen coefficient, (*k*_*s*_) was calculated according to Eq. (4) (Rowe 1992) and presented in Table 3.

4 $$\frac{1}{{s_{20,w} }} = \frac{1}{{s_{20,w}^{0} }}\left( {1 + k_{s} c} \right)$$

**Table 3:** Summary of sedimentation velocity analysis of fungal mannan samples, including sedimentation coefficients corrected for solvent conditions and extrapolated to infinite dilution

Sample	Peak	Fraction (%)	$s_{20,w}^{0}$ (S)	*k*_*s*_ (ml g^−1^)
FM-1	1	100	5.6 (±0.4)	470 (±140)
FM-2	1	53	5.3 (±0.2)	10 (±200)
	2	47	9.1 (±0.1)	29 (±70)
FM-3	1	100	3.3 (±0.1)	137 (±65)
FM-4	1	92	4.3 (±0.1)	60 (±23)
	2	8	12.8 (±0.5)	N/D^a^

*Values in parentheses* represent standard error of the mean (S) or regression (*k*_*s*_)

^a^Peak 2 of FM-4 was averaged (mean), no linear regression was estimated

**Fig. 6:**
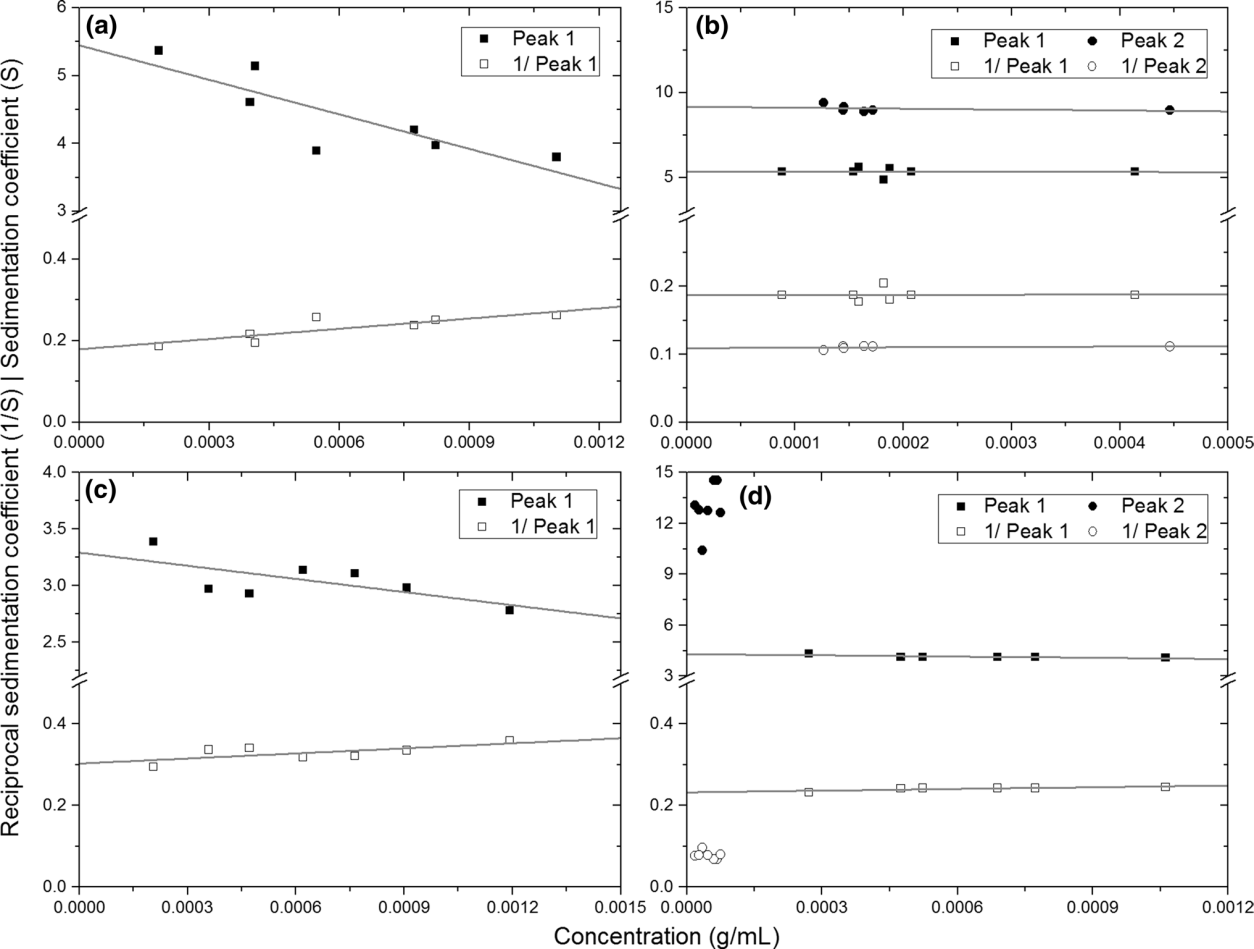
Extrapolations of integrated peaks from sedimentation velocity measured using linear regression (*grey line*) of native and reciprocal sedimentation coefficients against concentration of **a** FM-1; **b** FM-2; **c** FM-3; and **d** FM-4

Figure 6 shows that FM-1 (a) and FM-3 (c) have one component whereas FM-2 (b) and FM-4 (d) had two different components, based on distributions in Fig. 5. It can be observed that these mannan samples show little dependence of concentration on sedimentation coefficients over the small concentration range studied. The second peak from FM-4 showed no definite trend, thus the values were averaged (gradient = 0) to yield $s_{20,w}^{0}$ and no *k*_*s*_ estimated because of the very low concentration range.

There is little difference between extrapolated values of native and reciprocal sedimentation coefficients and similar errors. Values ranged between 3 and 6 S, for the smaller peak. Gralen coefficients differed more between the two methods and generally yielded higher error in the reciprocal regression. The single-peak samples were in the 100 range—118–310 ml g^−1^, whereas the two-peak samples were in the tens range—14–56 ml g^−1^. This indicates that the single-peak samples have a higher non-ideality than the two-peak systems, however the Gralen coefficients for FM-2 and FM-4 may be affected by the Johnston-Ogston effect (1946).

### Molar mass distributions from the extended Fujita approach

**Fig. 7:**
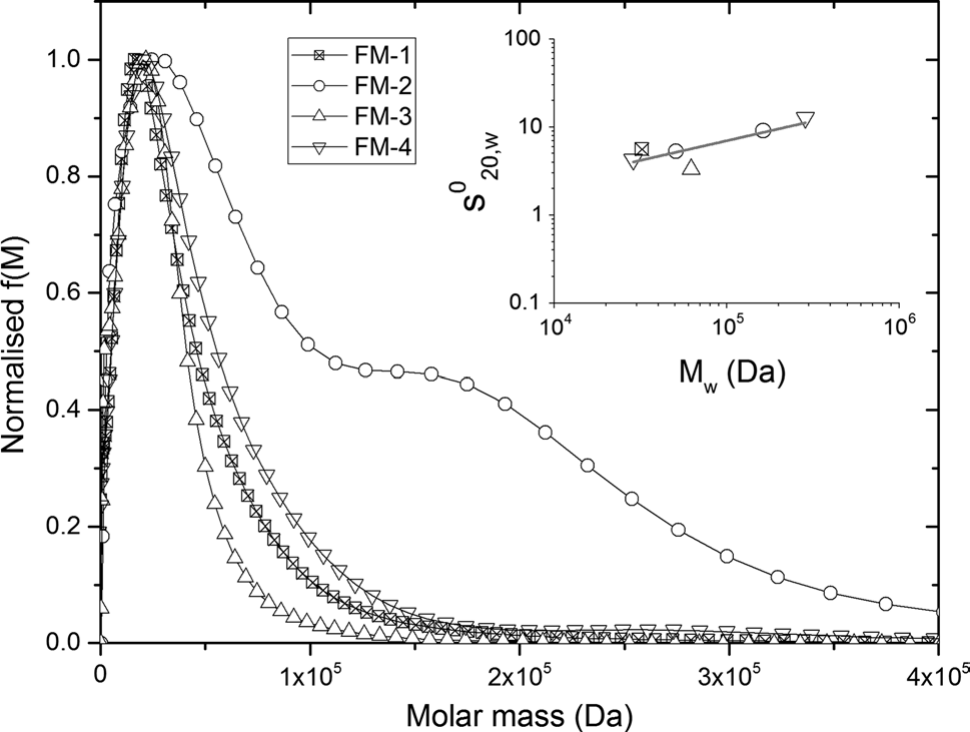
Extended Fujita approach (normalized *f*(*M*) vs. *M*) for FM-1, FM-2, FM-3, and FM-4. *Inset* is Mark–Houwink–Kuhn–Sakurada plot of six peaks from AUC-SV and AUC-SE (MULTISIG)

Combining information from AUC-SV (sedimentation coefficients) and AUC-SE (molecular weights) allow for the calculation of power-law scaling factors. The slope of a double logarithmic plot of sedimentation coefficients against molar mass yields the Mark–Houwink–Kuhn–Sakurada (MHKS) sedimentation shape factor *b* (Eq. 5).

5 $$s_{20,w}^{0} = \kappa_{s} M^{b}\quad {\text{or}}\quad M = \left( {\frac{{s_{20,w}^{0} }}{{\kappa_{s} }}} \right)^{{{\raise0.7ex\hbox{$1$} \!\mathord{\left/ {\vphantom {1 b}}\right.\kern-0pt} \!\lower0.7ex\hbox{$b$}}}}$$

where *κ*_*s*_ is an intercept constant (not to be confused with the Gralen coefficient, *k*_*s*_). This factor ranges between ~0.15 for a ‘rod’ to ~0.67 for a ‘sphere’ and 0.5 is a ‘random coil’. A previously published study showed that the MHKS shape factor was 0.43—this equates to a random coil, but on the ‘stiffer’ end of the scale (Pavlov et al. 1992). The inset of Fig. 7 shows results from this investigation plotted on a double-logarithmic scale. These data points include individual peaks from FM-2 and FM-4, with peak average molar masses obtained from MULTISIG. The gradient was 0.446 (±0.154), with a *κ*_*s*_ of 4.11 (±3.38) × 10^−2^, which is well within experimental error of the literature value of 0.43, corresponding *κ*_*s*_ of 5.09 (±0.01) × 10^−2^. The agreement of the *b* from Pavlov et al. (1992) and the present study justifies the assumption we have made that *M*_*w*,app_ measured at 1.0 mg ml^−1^ is ~*M*_*w*_.

The extended Fujita approach is a method for yielding molar mass distributions from g(*s*) distributions from AUC-SV (Harding et al. 2011). The original method assumed that the macromolecule was a random coil, which meant that the sedimentation coefficient was directly proportional to the square root of the molar mass:

6 $$s_{20,w}^{0} = \kappa_{s} M^{0.5} \;{\text{or}}\;M = \left( {\frac{{s_{20,w}^{0} }}{{\kappa_{s} }}} \right)^{2}$$

Harding et al. (2011) extended this approach to all conformational types using the general power relation (Eq. 6). To transform a distribution of sedimentation coefficients *g*(*s*) vs. *s* to a distribution of molar masses *f*(*M*) vs. *M* the following transformation equations are used:

7 $$f\left( M \right) = g\left( s \right)\frac{{{\text{d}}s}}{{{\text{d}}M}}$$

where

8 $$\frac{{{\text{d}}s}}{{{\text{d}}M}} = b\kappa_{s}^{{{1 \mathord{\left/ {\vphantom {1 b}} \right. \kern-0pt} b}}} s^{{{{\left( {b - 1} \right)} \mathord{\left/ {\vphantom {{\left( {b - 1} \right)} b}} \right. \kern-0pt} b}}}$$

In this instance, ls-g*(*s*) analysis performed by SEDFIT can be used to substitute *g*(*s*) in these equations, with *b* and *κ*_*s*_ calculated from the double logarithmic plot of sedimentation coefficient and *M*_*w*_. Using Eq. (5) to modify the abscissa and Eqs. (7) and (8) to modify to ordinate, the (normalized) *f*(*M*) vs. *M* plots are shown in Fig. 7. The distributions show a reduction in peak height for FM-2 peak 2 compared to ls-g*(*s*) from AUC-SV, but correlates more with the *g*(*M*) vs. *M* distribution from MULTISIG AUC-SE. Peak 2 for FM-4 is also reduced, which is less consistent with MULTISIG AUC-SE, which is likely an over-simplification of species present in solution. FM-1, FM-2, and FM-4 all showed overlapping peaks around 2–3 × 10^4^ g mol^−1^ with broad distributions leading to ~1.5 × 10^5^ g mol^−1^, consistent with information from AUC-SE.

Polydispersity indices calculated from these distributions were significantly higher than those for MUTLISIG and SEDFIT-MSTAR, with approximately 50–60 % higher estimations for *z*/*w*. The extended Fujita approach is based on *κ*_*s*_, which was calculated with a high standard error (4.11 (±3.38) × 10^−2^, ±82 %) from Fig. 7 inset. From Eq. (8), it is shown that *κ*_*s*_ has a large influence on the calculation of d*s/*d*M* and thus on the spread of the distribution. The high apparent polydispersity from *f*(*M*) can therefore be attributed to this high error in the *κ*_*s*_ value obtained from Fig. 7 inset. Although, in this instance, the extended Fujita approach provided a poor estimation of polydispersity, it did yield accurate estimates for molar mass and heterogeneity.

### Non-ideality

On the subject of non-ideality, concentration dependence was directly measured using AUC-SV to yield *k*_*s*_ values, which showed very low non-ideality for all four mannans. AUC-SE did not directly measure concentration dependence, however SEDFIT-MSTAR does provide indications where non-ideality significantly impacts the result. For example, discrepancies between consensus hinge-point and extrapolated M*(*r*) values, poor *c*(*M*) fits and negatively sloping point-average plots would all indicate the presence of significant levels of non-ideality—however they were not observed in the samples analyzed in this investigation. There would also be a poor correlation between the MHKS value obtained from the literature and these mannans. We can therefore say with confidence that there is a negligible effect of non-ideality present, but the values we report are likely to be slight under-estimates from the true values, although not significantly so.

## Conclusions

Four fungal mannan samples were probed for their molar mass and sedimentation coefficients using well-established techniques in the field of polysaccharide characterization.

The two independent analysis techniques used for AUC-SE (SEDFIT-MSTAR and MULTISIG) showed very good agreement. The obvious advantage for MULTISIG was the ability to yield molar mass distributions, particularly insightful for FM-2 and FM-4, as well as reliable values for *M*_*n*_ and *M*_*z*_, but at a cost of processing time (typically, a set of 20 fits can take between 20 and 30 min). Compared to this, SEDFIT-MSTAR is a much faster analysis method (a fit taking no more than 10 s) providing reliable and accurate weight and *z*-average molecular weights but with limited information of molar mass distribution. Combining information from AUC-SE and AUC-SV provided conformation information consistent with previously established results.

These methods (MSTAR, MULTISIG, extended Fujita) have shown a rapid assay for determining the molecular weight distribution of mannans, although they can also be adapted for use with other biopolymer solutions, including polysaccharides, DNA, and proteins—particularly relevant for the characterization of intrinsically disordered proteins. The significance of this assay is the characterization of a polydisperse, heterogeneous biopolymer with significance in various healthcare applications.
